# A Reversible Cause of Skin Hyperpigmentation and Postural Hypotension

**DOI:** 10.1155/2013/680459

**Published:** 2013-06-11

**Authors:** Rabia Cherqaoui, Mehreen Husain, Sujay Madduri, Pamela Okolie, Gail Nunlee-Bland, James Williams

**Affiliations:** ^1^Department of Internal Medicine, Howard University, Washington, DC 20060, USA; ^2^Department of Family Medicine, York Hospital, York, PA 17403, USA

## Abstract

Vitamin B_12_ deficiency results in neuropsychiatric, hematologic, gynecologic, cardiovascular, and cutaneous manifestations. It is seen most commonly in the elderly, malabsorption diseases  (>60% of all cases), vegans, and vegetarians. Manifestations of pernicious anemia may be similar to Addison disease and may lead to a misdiagnosis. Herein, we report two cases of vitamin B_12_ deficiency in which clinical features shared many similarities with Addison disease. Both patients presented with progressive darkening of hands and postural hypotension that reversed with replenishment of vitamin B_12_. Vitamin B_12_ deficiency should be considered in patients presenting with skin lesions especially with other coexisting autoimmune diseases.

## 1. Introduction

Vitamin B_12_ is a water soluble vitamin, present in various forms: cyanocobalamin (vitamin B_12_), hydroxocobalamin (vitamin B_12a_), aquacobalamin (vitamin B_12b_), nitritocobalamin (vitamin B_12c_), 59-deoxyadenosylcobalamin (coenzyme B_12_), and methylcobalamin (methyl B_12_). Its deficiency is a major health issue and a major diagnostic challenge; its presentation varies from being asymptomatic to affecting multiple organ systems. Some of the better studied and better known manifestations are hematologic such as macrocytic anemia, pancytopenia and neurological, such as orthostatic hypotension, paraesthesias, and abnormal gait [1, 2]. Some of the lesser known manifestations are cutaneous such as skin hyperpigmentation, stomatitis, and hair and nail changes. These may be important to recognize as early treatment may prevent potentially irreversible complications.

## 2. Case Presentation

### 2.1. Case 1

A 40-year-old African American woman with history of myasthenia gravis, Hashimoto's thyroiditis stable on thyroxine replacement, and childhood asthma was seen in the outpatient clinic for numerous complaints including fatigue, multiple syncopal episodes, and diffuse darkening of the palms of both hands for the past 3 to 4 months. There was no rash or dermatitis preceding the onset of hyperpigmentation. 

She is a nonvegetarian and reports occasional alcohol use but no smoking or illicit drugs. She has a family history of type 2 diabetes mellitus, heart disease, hypertension, autoimmune disorders, and hypothyroidism.

**Figure 1:**
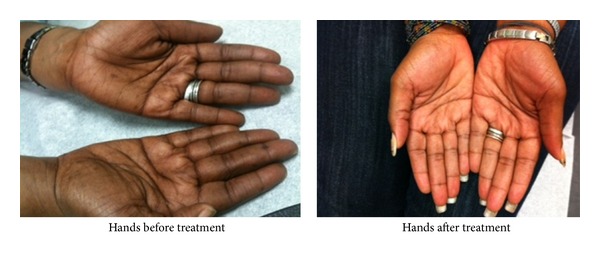


Physical examination showed a well-developed woman in no distress. Her blood pressure was 113/88 sitting, 105/76 standing, temperature of 37° Celsius, pulse of 92 (sitting), 112 beats/minute (standing), and respiratory rate of 16 breaths/minute. She had pale mucus membranes. The respiratory, cardiovascular, neurological, and abdominal examinations were unremarkable. Axillary and pubic hairs was intact. Her extremities showed diffuse hyperpigmented, nonpruritic, and macular lesions on the palms and soles bilaterally (Figure 1).

Pertinent laboratory studies were as noted in Table 1. Cosyntropin stimulation test was performed to evaluate for possible adrenal insufficiency in view of history of other underlying autoimmune disorders. Patient responded appropriately with serum cortisol of 7.1 *μ*g/dL at baseline, 22.9 *μ*g/dL at 30 minutes, and 27.3 *μ*g/dL 60 minutes after cosyntropin administration. Further investigations revealed positive blocking antibodies to intrinsic factor, and diagnosis of B_12_ deficiency due to classical pernicious anemia was made.

**Figure 2:**
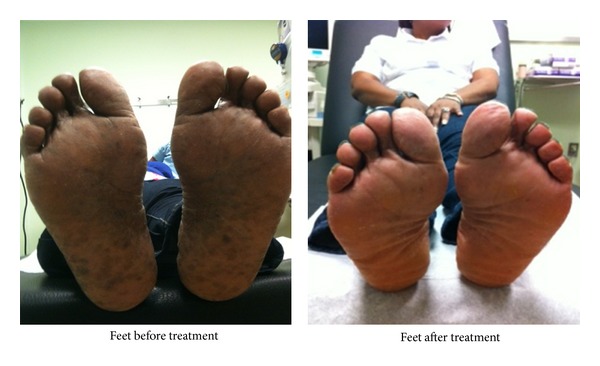


The patient was started on 1,000 *μ*g injections intramuscularly daily for 7 days followed by once a week and once a month for one year thereafter. Vitamin B_12_ level was repeated at 4 and 8 months with values of 269 pg/mL and 412 pg/mL, respectively. There was marked improvement in fatigue, malaise, dizziness and resolution of syncope. The hyperpigmentation in hands and feet as shown in Figures 1 and 2 below resolved with no recurrence.

### 2.2. Case 2

A 65-year-old African American woman was rushed to the hospital by ambulance following an episode of syncope at a community supermarket. She regained consciousness within a few minutes. There was no tongue biting, urinary incontinence, or postictal confusion. She reported some premonitory symptoms of warmth and sweating and gave a history of light-headedness, weakness, and fatigue over the last 2 weeks. She denied palpitations, chest pain, shortness of breath, fever, dizziness, or visual changes. She denied headache or weakness.

Over the last couple of months, she had multiple episodes of fall but no loss of consciousness. The falls were increasing in frequency over the last 2 weeks. She lived alone and had a history of hypertension for almost thirteen years for which she was noncompliant with her medications. She did not smoke, drink alcohol, or use any illicit drugs. Her mother had hypertension and died at the age of 80 years from heart disease. 

On examination, her temperature was 37°C, the respiratory rate was 24 breaths per minute, and blood pressure while supine was 89/52 mm Hg, with a heart rate of 52 beats per minute; on standing, her blood pressure fell to 69/40 mm Hg, with a heart rate of 74 beats per minute. Her blood glucose level was 120 mg per deciliter. Oxygen saturation was 100 percent on 2 liters of oxygen via nasal cannula. 

**Figure 3:**
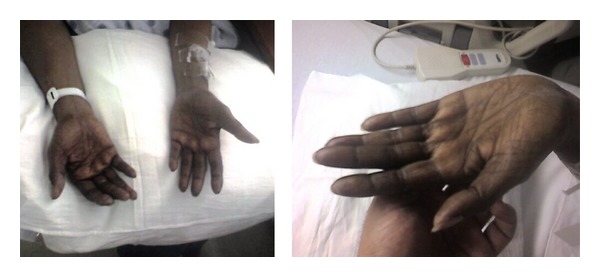


Physical evaluation showed no signs of head trauma. Her respiratory, cardiovascular, and abdominal examinations were unremarkable. Neurological examination showed decreased sensation to light touch in a stocking and glove distribution. Upper limb reflexes were brisk and lower limb reflexes were absent. Vibratory sensation and position sense were decreased in both upper and lower limbs. An extensor plantar response was noted bilaterally and Romberg's test was positive. There was hyperpigmentation of both palms with dark discoloration of the nails (Figure 3). Axillary and pubic hair was normal in distribution and density. She was admitted to the hospital for further investigation and workup.

Contrast computed tomography (CT) of the head showed no obvious lesion. An electrocardiogram revealed sinus bradycardia at a rate of 50 beats per minute with no ST or T wave changes. A 24-hour holter monitoring was performed which revealed sinus bradycardia without other arrhythmias.

Her laboratory studies were as shown in Table 2.

Serum alcohol levels were undetectable and urine toxicology was negative. Esophagogastro-dudodenoscopy was performed and the gastric biopsy was suggestive of chronic atrophy of the body and fundus. The endoscopic findings were further reinforced by high serum levels of antibodies to intrinsic factor and gastric parietal cells confirming malabsorption of vitamin B_12_ due to lack of intrinsic factor. Thus, the diagnosis of pernicious anemia was established.

The patient was started on intramuscular injection of vitamin B_12_ (1,000 *μ*g) daily for one week, then weekly for one month. She was later lost to followup, and posttreatment evaluation of the skin hyperpigmentation and neurological evaluation could not be done.

## 3. Discussion

Studies show a prevalence of cobalamin deficiency around 20% (ranging from 5 to 60% depending on parameters for cobalamin) in industrialized nations [3]. The primary source of vitamin B_12_ is derived from animal products such as dairy products, meat, eggs, fish, and shellfish. Vitamin B_12_ deficiency becomes important in high-risk populations such as the elderly, patients with either malabsorption diseases as in gastric and ileal resection or gastric mucosal disease resulting from either an autoimmune process or from gastric atrophy, pancreatic insufficiency, coeliac disease, Crohn's disease, ileitis, vegetarians and long-term use of proton pump inhibitors [4], histamine (H2) receptor antagonists [5], or biguanides [6, 7]. 

In pernicious anemia, the gastric parietal cells are targeted by the autoimmune process. Hence, the production of intrinsic factor is affected leading to defective absorption of vitamin B_12_.

 The effects of vitamin B_12_ deficiency on the hematologic, nervous, and gastrointestinal systems are well studied. It has been implicated as a cause of orthostatic hypotension [8], mood disorders [9], dementia, psychosis, depression, myocardial infarction, stroke [10], and retinal hemorrhages [11]. Orthostatic hypotension is considered a neurological manifestation of vitamin B_12_ deficiency and clearly relates to the underlying autonomic neuropathy [12]. 

Historically, Thomas Addison described pernicious anemia and Addison's disease. There is a debate about whether Thomas Addison himself mistakenly reported both clinical entities in his 1855 monograph on adrenal disease [13]. Both disease entities share several clinical features particularly asthenia, gastrointestinal symptoms, hyperpigmentation, and hypotension. Consequently, it may be worthwhile to consider vitamin B_12_ deficiency and determine serum B_12_ level in all the patients with symptoms suggestive of Addison disease such as hyperpigmentation and orthostatic hypotension. Early diagnosis can limit morbidity and inappropriate workups. It is also important to remember that, although uncommon, both diseases can coexist in the same patient in the context of “polyglandular autoimmune syndromes.”

The dermatologic manifestations, first described in 1944 by Dr Bramwell Cook [14], are often overlooked. The cutaneous manifestations described in the literature include but are not limited to skin hyperpigmentation, hyperpigmentation of surgical scars [15], and mucus membrane of the vulva [16], vitiligo, recurrent angular stomatitis, and hair changes. In a study of 63 patients by Aaron et al. glossitis (31%) was the most common mucocutaneous presentation, followed by skin hyperpigmentation (19%), hair changes (9%), angular stomatitis (8%), and vitiligo (3%) [16]. Characteristically, hyperpigmentation is observed in the oral mucosa and over the dorsum of hands and feet, with accentuation over the interphalangeal joints and terminal phalanges [17], occasionally in the creases of palms and soles, rarely on lower extremities. The nails may have hyperpigmented streaks with pale nail beds. The mechanism of hyperpigmentation is unknown although increased melanin synthesis has been suggested [18]. Premature graying of scalp hair has also been described [19]. In primary adrenal insufficiency, hyperpigmentation of mucous membranes and skin, particularly of body creases, pressure points, areolas, anogenital area, and scars, is considered a cardinal manifestation of this disease. The hyperpigmentation seen in Addison disease has been linked to increased melanocyte-stimulating hormones (alpha and beta MSH) and adrenocorticotrophin (ACTH).

An understanding of the enzymatic reactions dependent on vitamin B_12_ helps appreciate the mechanisms involved in the development of neurological complications. Two principal enzymatic reactions require vitamin B_12_ as a key cofactor: the mitochondrial conversion of methylmalonyl-CoA to succinyl-CoA and the cytoplasmic methyl transfer reaction that converts homocysteine to methionine. The latter reaction is accompanied by the conversion of methyltetrahydrofolate to tetrahydrofolate, a precursor for purine and pyrimidine synthesis. The interruption of this step due to vitamin B_12_ deficiency leads to impaired synthesis of DNA and a megaloblastic maturation pattern of hematopoietic cells [20].

Methionine is converted to S-adenosylmethionine (SAM) which methylates neurotransmitters and phospholipids [21]. Vitamin B_12_ deficiency interferes with production of choline and choline-containing phospholipids, oligodendrocyte function, and myelin basic protein methylation. The neurological damage usually begins with demyelination, followed by axonal degeneration and eventually irreversible axonal death. The neurological damage is reversible when replacement therapy is initiated early. Our patient (case 2) had classical presentation of subacute combined degeneration of the cord as indicated by the positive Babinski and Romberg sign.

Our cases highlight the fact that the clinical presentation of vitamin B_12_ deficiency may be similar to Addison disease and patients can present with primarily cutaneous symptoms that improved dramatically with vitamin B_12_ replacement. Very few other cases to our knowledge have had similar presentations. This is to emphasize that early recognition of mucocutaneous symptoms may aid to prevent severe irreversible neurological complications in the future [22].

## 4. Conclusion

Vitamin B_12_ deficiency may be more common than generally thought and is most likely under-diagnosed due to varied and unusual presentations. It may be worthwhile to consider vitamin B_12_ deficiency and determine serum B_12_ level in all the patients with symptoms suggestive of Addison disease such as hyperpigmentation and orthostatic hypotension. Early diagnosis can limit morbidity and inappropriate workups. Hyperpigmentation associated with vitamin B_12_ deficiency is completely reversible with treatment. 

## Figures and Tables

**Table 1:** Summary of patient's laboratory results.

Investigation	Pretreatment	Posttreatment	Reference range*
Hemoglobin (gm/dL)	10.9	10.8	12–16 gm/dL
Hematocrit %	31.6	31.1	43.3%–46.6%
Mean corpuscular volume: MCV (fL)	113	86.4	80–100 fL
Serum vitamin B_12_ (pg/mL)	67	412	200–950 pg/mL
Thyroid-stimulating hormone: TSH (milli-int units/L)	1.36		0.4–4.0 mIU/L
Free thyroxine (microgm/dL)	1.47		0.7–2.0 mcg/dL
Plasma morning cortisol (microgm/dL)	7.1		
Post-adrenocorticotropic hormone (ACTH) plasma cortisol (microgm/dL)	27.3		Normal > 18

*Reference values are affected by multiple variables, including patient population and laboratory method used.

**Table 2:** Summary of patient's laboratory results.

Investigation	Patient value (pretreatment)	Reference range*
Hemoglobin	10.5 gm/mL	12–16 gm/dL
Hematocrit	30.4	43.3%–46.6%
MCV	121 fL	80–100 fL
Platelet	133 × 10^9^/L	177–406 Th/cm
Total white count	4.9 × 10^9^/L	3.2–10.6 × 10^9^/L
Peripheral smear	Macrocytosis	—
Serum vitamin B_12_	55 pg/mL	200–950 pg/mL
Serum folate		
Plasma morning cortisol mcg/mL	10.24	
Post-ACTH plasma cortisol	41.11	Normal > 18

*Reference values are affected by multiple variables, including patient population and laboratory method used.
